# Implementation of tele-geriatricmental healthcare for rural veterans: factors influencing care models

**DOI:** 10.3389/frhs.2024.1221899

**Published:** 2024-11-29

**Authors:** Christine E. Gould, Lynsay Paiko, Chalise Carlson, Marika Blair Humber, Ranak Trivedi, Julie Filips, A. Denise Savell, Althea Lloyd, Amanda D. Peeples

**Affiliations:** ^1^ Geriatric Research, Education and Clinical Center (GRECC), VA Palo Alto Health Care System, Palo Alto, CA, United States; ^2^ Department of Psychiatry and Behavioral Services, Stanford University School of Medicine, Palo Alto, CA, United States; ^3^ Psychology Service, VA Palo Alto Health Care System, Palo Alto, CA, United States; ^4^ Center for Innovation to Implementation, VA Palo Alto Health Care System, Palo Alto, CA, United States; ^5^ VISN 23 Clinical Resource Hub, Minneapolis VA Health Care System, Minneapolis, MN, United States; ^6^ VISN 7 Clinical Resource Hub, Atlanta VA Health Care System, Atlanta, GA, United States; ^7^ Center for Health Equity Research and Promotion, Corporal Michael J. Crescenz VA Medical Center, Philadelphia, PA, United States

**Keywords:** telehealth, veterans, older adults, implementation, CFIR framework

## Abstract

**Introduction:**

Aging rural veterans have limited access to geriatric mental health services. The establishment of Veterans Health Administration (VHA) regional telehealth hubs, or Clinical Resource Hubs (CRHs), has the potential to improve access to specialist care via telehealth delivered across healthcare systems within each VHA region. We used the Consolidated Framework for Implementation Research (CFIR 1.0) to examine variations in the tele-geriatric mental health (tele-GMH) care models being used in four CRHs.

**Methods:**

We interviewed 11 CRH geriatric mental health providers and 12 leaders to (1) characterize the models of care, (2) identify factors in their region that support tele-GMH, (3) identify factors underlying model adaptations, and (4) learn about barriers and facilitators during implementation. The interviews were analyzed using a combination of CFIR-based coding and rapid qualitative analysis.

**Results:**

The services used multiple telehealth modalities; their care delivery approach ranged from consultative to continuity services. Aspects of the inner setting, specifically structural characteristics, implementation climate, and implementation readiness, influenced the model that each CRH implemented. Barriers were largely related to inner setting structural characteristics. Facilitators highlighted the importance of planning, iteration, and engaging stakeholders during implementation.

**Conclusion:**

Tele-GMH models varied in approach, tailoring their services to fit inner setting characteristics. Barriers and facilitators remained consistent across regions. Attending to inner setting characteristics, ongoing process improvement, and nurturing relationships with stakeholders is critical throughout the implementation of a tele-GMH program. Future research should examine the impact of the varied care delivery models on quantitative outcomes, including metrics related to access and healthcare utilization.

## Introduction

More aging veterans reside in rural areas (55%) than urban areas (1). Veterans residing in rural areas are 20% more likely to die by suicide (2) and 70% less likely to utilize any mental health (MH) services compared with non-rural veterans (3). Moreover, rural older adults have higher rates of chronic health problems (4) and are less likely to access MH services (5). Thus, both rurality and age serve as risk factors for higher rates of suicide and medical and neurocognitive conditions. These complex needs often warrant specialty geriatric MH care.

Aging rural veterans may face numerous barriers to accessing MH care. Both general MH professionals and geriatric MH specialists are primarily located in urban centers (6, 7). Only 15% of Veterans Health Administration (VHA) medical centers are in rural areas, where most older veterans (≥65 years) reside (8). Long travel times for rural veterans to reach medical centers and the limited availability of VHA staff to visit homebound rural veterans impede access to MH care (9).

Video telehealth services have proliferated rapidly in the VHA, increasing access to mental healthcare through clinical video telehealth (CVT) from hub to spoke sites (10) and through video to the home using VHA-provided devices (11). However, these innovations do not specifically address the need for specialty MH services, such as access to geriatric psychiatrists (7). The VHA has developed regional telehealth hubs, called Clinical Resource Hubs (CRHs), that provide telehealth services across multiple healthcare systems in a VHA region [Veterans Integrated Service Networks (VISNs)], often spanning multiple states (12). CRHs provide specialty care programs, including a tele-geriatric mental health (tele-GMH) service that was initiated in 2018 and is focused on providing geriatric psychiatry consultations with time-limited follow-up (13). This original model focuses on timely access to experts, when needed, for diagnostic clarification and both pharmacological and non-pharmacological interventions. The present program evaluation examines the naturalistic expansion and regional adaptation of this model in three additional CRHs. The tele-GMH specialty care models implemented in each VISN vary in composition, roles, and dedicated time allotted to the tele-GMH service. Our evaluation examines the implementation and adaptation of this intervention using the Consolidated Framework for Implementation Research (CFIR 1.0) (14). The CFIR was selected to help organize the constructs that led to the original tele-GMH model. Further, the CFIR provides a framework for characterizing the regional (i.e., inner setting) factors that contributed to leaders’ decisions regarding adaptations made to the tele-GMH services and provides a practical approach to considering the barriers to and facilitators of implementation.

## Methods

This evaluation presents a qualitative analysis of key stakeholder interviews with leaders and tele-GMH providers during an ongoing naturalistic observation of the expansion of tele-GMH services in the CRH program. This program evaluation was determined by the Stanford University IRB to be non-human subjects research.

In total, 23 key stakeholders (*n* = 12 VISN or CRH leaders, *n* = 11 tele-GMH providers) participated in interviews between October 2020 and February 2023. The interviews were conducted in two phases. In Phase 1 (October 2020 to May 2021), seven interviews were conducted with one tele-GMH provider and six VISN leaders from the originating site of the tele-GMH service. In Phase 2 (February 2022 to February 2023), interviews were conducted with 10 tele-GMH providers to characterize their models of care and with 6 MH section leaders or directors in the CRHs where tele-GMH services were implemented to identify the factors that contributed to their region’s ability to support the services and the factors underlying their model adaptations. Using CFIR 1.0, we focused on each VISN's *inner setting* (e.g., structural characteristics, culture, and readiness for implementation), *outer setting* (e.g., patient needs and resources), the fit with the *intervention* (e.g., intervention source, complexity, and trialability), and the *process* (e.g., planning, and engaging).

### Phase 1 interviews and analysis

Interview guides were developed using questions from the CFIR 1.0 interview guide that focused on the four aforementioned domains. The interviews were transcribed verbatim, excerpted, and then coded using the CFIR codebook. Inductive codes were added to capture barriers, suggestions, and leadership decision points that influenced the type of model to pursue. A consensus approach was used for code application. Discrepancies were discussed and resolved in a team-based analysis.

### Phase 2 interviews and analysis

Interviews were conducted with tele-GMH providers (*n* = 10) who were asked about the services offered, common referral reasons, settings for referred patients, collaboration on cases, and overall approach to care (e.g., consultative or continuity). The responses were summarized and used to draft descriptions of the models. The interviews with leaders focused on understanding regional needs, decision points leading to the selection of a model, barriers to/facilitators of their VISN's implementation, and the service's impact on stakeholders.

These interviews were summarized using rapid qualitative analysis techniques (15–17). We summarized the transcripts into key domains and then reviewed the domains to identify themes using a team-based approach.

## Results

### Phase 1 interviews

Interviewee characteristics are shown in Table 1. The original model (VISN A) consisted primarily of consultative care by a geriatric psychiatrist with limited follow-up appointments. Two key CFIR domains underlie the development of this model: *intervention characteristics* and *inner setting*.

**Table 1:** Interviewee characteristics.

	Category	N	%
Phase 1 (*n* = 7)			
Discipline	Administration (non-clinician)	2	28.6
	Physician	5	71.4
Leadership setting	Mental health	3	43
	Geriatrics	2	28.6
	Other	1	14.3
	N/A^a^	1	14.3
VA region	VISN A	7	100
Phase 2 (*n* = 16)			
Discipline	Physician	6	37.5
	Psychologist	9	56.3
	Other	1	6.3
Leadership setting	Mental health	6	37.5
	N/A^a^	10	62.5
VA region	VISN A	2	12.5
	VISN B	5	31.3
	VISN C	5	31.3
	VISN D	4	25.0

Note: ^a^One interviewee in phase 1 and 10 interviewees in phase 2 were in clinical roles only.

### Intervention characteristics

The *intervention characteristics*, that is, the key attributes of the tele-GMH service [for details see Gould et al. (13)], were deemed to have an advantage over other possible interventions based on the characteristics of the *inner setting*. The leaders discussed the *complexity* of the intervention in that the geriatric psychiatrist serves multiple care settings, ranging from outpatient to residential/inpatient care, and works with both individual providers and healthcare teams. The leaders emphasized the *relative advantage* of telehealth consultation in providing psychiatric coverage to the wide catchment area in their region:

“Our motto is a consultative model instead of a follow-up and take-care-of model because it's just too wide of an area to take care of and follow-up with [patients]. So, that's why [the psychiatrist] does not prescribe, [the psychiatrist] makes recommendations, and of course the provider can always re-consult.” (Leader 3).

The leaders also discussed the advantage of consultation, explaining that patient panels in continuity care would fill up and limit access to the specialist. Moreover, the management of medications, clinical reminders, and lab orders across multiple healthcare systems was deemed to be a “logistical nightmare.”

### Inner setting

*Inner setting* encompasses an examination of the regional factors, including culture, structure, and indicators that signal a preparedness to commit to the implementation of an intervention. The leaders suggested that the region's *culture* (VISN A), which specifically values innovation and staff dedication to geriatrics and MH, led to the flourishing of this consultation-based model.

Leaders described the region's *structural characteristics* as a key factor influencing the design of the tele-GMH service. Leader 5 explained,

“One of the things that is somewhat unique is the rurality, kind of how geographically spread-out we are … it's probably 300+ miles wide in a lot of places, so we have [an outpatient clinic] that is … 300 miles from my office.”

The combination of the region's *structural characteristics* and changes in staffing (i.e., the loss of a psychiatrist at a VHA nursing home) led to an increased *readiness for the implementation* of telehealth services. This intervention, while highly complex, was able to succeed due to *leadership engagement* helping delineate the boundaries of care (e.g., medication recommendations rather than ordering medications) provided by the geriatric psychiatrist. A leader described the complexity of this model and how their VISN was able to navigate these challenges:

“It has to have leadership support at the various health care systems to accept this consultative model versus the traditional panel model. And then, it has to have senior level support because as a consult service, there are fluctuations in productivity. It's a consult, you don't have a regular panel that you can schedule every 30 min, some days or some weeks there are numerous consults, and then some weeks there are not.” (Leader 3).

### Phase 2 interviews

Due to the success of the original model, variations of this model began to be developed in different regions (see Table 2). All models used multiple telehealth modalities including e-consultation, video-to-home telehealth [VA Video Connect (VVC)], CVT to VHA sites, and telephone visits.

**Table 2:** Geriatric mental healthcare models used by VISNs.

Model types	Consultative with limited follow-up	Consultative “Treat-and-Return”	Continuity and **tele-**neuropsychology assessment	Hybrid (50% consultation/50% continuity)
Region	VISN A	VISN B	VISN C	VISN D
Description	• Comprehensive, prompt consultation consistent with consultation liaison approach • Time-limited follow-up (including psychotherapy)	• Delivers episodes of care to stabilize symptoms with the goal to return to regular providers or connecting to general mental health • Shared intake appointments with 2 or more providers • Medication prescribing, behavior plans, psychotherapy including caregiver support	• Continuity geriatric psychiatry services • Continuity psychotherapy • Tele-neuropsychology assessments with targeted batteries (≥4 h)	• Consultation with CLC and outpatient clinics • Continuity geriatric psychiatry services • Geriatric consultation consistent with AFHS/4Ms
Disciplines on team	• Geriatric psychiatrist • Geropsychologist • RN case manager	• Geriatric psychiatrist • Geropsychologist • Clinical pharmacy specialist • RN case manager	• Geriatric Psychiatrist • Geropsychologist • Neuropsychologist	• Geriatric Psychiatrist • Geriatrician
Care settings	• CLC • HBPC • Outpatient • Inpatient	• Outpatient • HBPC	• Outpatient	• CLC • HBPC • Outpatient

RN, registered nurse; CLC, community living center; HBPC, home based primary care; AFHS, age-friendly care.

Note: Some teams have grown in the number of disciplines and number of individual providers included in the team since this evaluation was completed.

### Model variations and associated CFIR constructs

#### Consultation with time-limited follow-up

The VISN A model evolved from having a single geriatric psychiatrist consultant and nurse case manager (part-time) to a two-provider model with a geropsychologist in 2021.

Responding to the patient needs (*outer setting* and *patient needs and resources*) and aspects of the *inner setting* described by the leaders (Phase 1) drove the expansion of the consultation-based model to include time-limited psychotherapy and assessments (e.g., cognitive and capacity). One leader explained that “one of the things that perpetually came up for some of these more complicated cognitive cases that we really wanted that neuropsychology support for commenting on capacitance and that kind of thing. So, we're adding that” (Leader 11). Going on to discuss the hiring of a geropsychologist, the leader explained that they have received requests for geriatric psychotherapy services that are sensitive to the phase-of-life needs of older adults. The consultation model still primarily delivers consultative care with time-limited follow-up appointments and serves the majority of the VHA healthcare systems in the region.

#### Treat-and-return consultative model

This model, developed in VISN B, consists of a team-based consultative model that provides time-limited services to help stabilize patients, including developing behavioral plans and starting psychotropic medications as appropriate. Once patients are stabilized, the tele-GMH team transfers ongoing MH care back to the patient's primary care provider or another MH provider. Providers may re-consult as needed. The team includes a geriatric psychiatrist, an MH clinical pharmacist practitioner, and a geropsychologist who jointly conduct the intake assessment as a shared medical appointment. The clinical pharmacist or geriatric psychiatrist orders and manages the patient’s medications. The geropsychologist provides time-limited interventions that are focused on caregiver support, behavioral plans for managing distress behaviors, and time-limited psychotherapy.

Consideration of the *inner setting*, particularly the needs of local care teams*,* guided the implementation of tele-GMH in VISN B. The tele-GMH team conducted a needs assessment to guide the design of its approach. Following this assessment, the team tailored the service to the *inner setting*'s existing *structural characteristics* and the *readiness for implementation* to avoid the duplication of locally available services. They repeated this process for each spoke site as the service expanded throughout the region. The needs assessment led the team to design the model to help address local providers’ “exhaustion” in receiving time-consuming recommendations by instead implementing the recommendations themselves prior to returning the patient to the referring provider (Leader 7). The leader explained that through collaboration with the local team, the service provides timely care (e.g., conducting assessments and starting medications) when the local team is at capacity, thus reducing waiting times. The local providers responded well to the tele-GMH team's approach, thus demonstrating the *implementation climate’s compatibility* within the *inner setting*.

#### Continuity model

The continuity model in VISN C serves healthcare systems with a high need for outpatient MH care. VISN C leaders advocated for expanding CRH services to tele-GMH care based on the success of their established tele-neuropsychology service (*inner setting* and *implementation climate*) and the identified *patient needs* (*outer setting*) for geriatric MH care. In the continuity model, a geriatric psychiatrist and a geropsychologist provide ongoing MH services to their own panels of patients, independently conducting psychiatric diagnostic assessments and delivering ongoing services including medication management (geriatric psychiatrist), caregiver support, supportive psychotherapy, and evidence-based psychotherapy (geropsychologist). The geriatric psychiatrist and geropsychologist collaborate on treatment planning/care coordination for shared patients.

Decision points for the continuity model were related to the *inner setting* characteristics. One leader discussed the *structural characteristic* of frequent local provider turnover, which could become a barrier to generating sufficient consult requests. These contributing factors led the leaders to consider various approaches, and they determined that continuity care, based on the *relative advantage* of promoting and utilizing panel management compared with consultative care, was the best option.

#### Hybrid model (consultation/continuity clinic)

The hybrid model in VISN D consists of a half consultation and half continuity clinic. This model arose in response to the *inner setting* need for consultation in Community Living Centers (CLCs) and outpatient clinics and the need for continuity care for some medically complex veterans (*outer setting* and *patient needs and resources*). In this model, two geriatric psychiatrists provide consultation and continuity services at different spoke sites. The leaders explained that certain *structural characteristics*, namely, rurality and the availability of local geriatric care, contributed to the design. Leader 9 emphasized the region's abundance of personnel to deliver services, thus demonstrating the region's *readiness for implementation*:

“I think we are more resource rich in [VISN D] in terms of geriatric specialists. [We] have some distinct training programs in our home VA. So, we have providers with that training and expertise, and obviously there's a need. I know a lot of the sites we work [with] don't have specialists, don't have enough providers at all, and certainly not for some specialty needs.”

The hybrid approach fits with the tele-GMH providers’ preference to follow a veteran longitudinally, and given the lack of geriatric psychiatrists, consultation services maximize veterans’ access to specialty MH care. Leader 12 emphasized that their hybrid model is currently in progress and “evolving.”

“… it's been ad hoc, but some of it is acting as a psychiatrist for older veterans, often with memory disorders and then the other part of it is trying to actually help build out kind of specialty teams at sites where the psychiatrist works with an Advanced Practice Provider of geriatrics trained therapist and a Case Manager for kind of more complicated population [to] kind of be a member of that team as well for a site that can't get the geriatric psychiatrist.”

### Barriers to implementation

All the leaders described challenges in coordinating between multiple systems and sites due to the different processes and structures in place within the *inner setting*. The *structural characteristics*, such as existing patient services in the region, scheduling processes for telehealth, and variations in systems at each local spoke site, made navigating the care delivery system challenging. For some, the lack of resources and services available in rural areas limited the community referrals and recommendations that tele-GMH providers could make. The leaders discussed the necessity to communicate with local providers and services to not encroach on the territory of local services. One leader described managing this through the tele-GMH team's collaborative and supportive nature to “… not take over … or duplicate anything that was already in place” (Leader 7).

Leader 7 reported they had to come up with their own process for implementing appointments in different facilities “… because we're at every single [outpatient clinic] and every single facility, that's a lot of clinics … we've had meetings with the [telehealth technicians] trying to learn what their processes are, what the best way to get a room is when we need it.” As Leader 9 described, “We learn one system and then we start from scratch [in another system].” Others cited difficulties related to scheduling visits when a provider is credentialed at one VHA facility and the patient is at another facility where the provider is not credentialed. Within the hubs, *inner setting* indicators of *readiness for implementation* included having dedicated support staff, such as a scheduler and/or a grid manager, to facilitate the coordination of these complex scheduling needs. As Leader 8 stated, “This program would not function without having an embedded MSA [medical support assistant] dedicated.”

Finally, the leaders discussed the challenges of the *implementation climate* with regard to balancing the workload for consultants with competing demands from local, regional, and national leaders regarding bookability metrics. Others expressed concern about the substantial consultant workloads due to the ongoing need from the spoke sites for geriatric MH care and the possibility of consultant burnout. Some leaders discussed trusting their CRH tele-GMH providers and allowing them to implement the service as they see fit without substantial concerns about meeting productivity metrics.

### Facilitators of implementation

Facilitators were identified at multiple time points during the *process* of developing services, service rollout at each new site, and ongoing delivery of tele-GMH services. The leaders discussed aspects of *planning* that were part of their implementation strategies. Two leaders expressed an understanding of the burden on referring providers and emphasized the importance of streamlining the consult process by using an easy, brief consult request template. Another site refined its consult template during the rollout process so that referring providers could enter a request “… in about 2 min …” (Leader 8). Coordination with spoke sites is critical, especially for CVT services. The CRH leadership and administrative team members have specific roles in ensuring that the setup at new sites runs smoothly by tracking metrics and fixing issues. Strong spoke site leadership support and organized CVT staff (i.e., telehealth technologists) served as a strong practice model for subsequent sites. Moreover, the marketing of services by the CRH leaders and the tele-GMH providers themselves is critical to generating referrals. These relationships are built and maintained by attending team meetings, describing their services, sharing flyers, and engaging with referring providers one-on-one.

Also important to the *process* is *engaging* appropriate individuals to support the rollout of these tele-GMH services, including regional geriatric MH leaders, the formal implementation team (i.e., tele-GMH providers), and spoke site champions. Regional leaders initially helped implement the service by demonstrating support by co-signing the initiative and by recognizing that “… geriatrics is the biggest cost and biggest need right now …” (Leader 11). Some regional leaders served in an advisory capacity to the CRHs. Relationships with local spoke site leaders also facilitated the implementation. As CRH leaders continued to engage the regional leaders to increase awareness of the service, leaders “… nurture[d] the referral bases there …” (Leader 8).

The tele-GMH providers helped facilitate the implementation process through their initiative, creation of standard processes, and “… best in class …” (Leader 8) and “… phenomenal …” (Leader 7) clinical skills. Relationships developed at each new local site where tele-GMH is rolling out services provide the foundation for successful service delivery. For example, Leader 12 described the importance of multiple types of relationships and buy-in to facilitate the uptake and delivery of services:

“In general, good relationships with folks on the ground, persistence and trying, you know, feeling within the team, that we should keep on moving forward even if it seems like we're struggling with adoption, that's been valuable. Buy-in from telehealth staff is critical. Buy-in from nursing. I think having healthy relationships with the mental health service line there where they [act as] collaborators is always [helpful,] and flexibility on everybody's part.”

Others noted that relationships with specific clinical services, programs, and individual providers, such as with a memory clinic provider or with the Suicide Prevention Program, help create familiarity with the sites, thus driving referrals.

## Discussion

Guided by the CFIR domains of *intervention characteristics*, *inner setting*, *outer setting*, and *process*, our findings identify key constructs that underlie the development and implementation of tele-GMH services in four VISNs. The findings suggest that tele-GMH services can be flexibly developed and applied to help fill geriatric MH service gaps for rural veterans.

The models of care varied in how they targeted *patient needs and resources* within each VISN. Decision points guided the tailoring of each model to specific *inner setting* needs. Notably, the models developed organically based on these needs, which resulted from the *structural characteristics* of the region, such as the need to cover distance in remote clinics or facilities, the *implementation climate*, or the *readiness for implementation*. Some VISNs had more available resources, such as hiring geriatric MH providers for CRH positions, whereas other VISNs faced substantial turnover of local providers within the healthcare systems. These findings fit with the broader CRH program in its regional organization, which allows for staffing resources to be directed to local sites with the greatest need (12). Our work extends the broader CRH evaluations to specialty care approaches by identifying the facilitators, barriers, decision points, and relative advantages of each model. However, future research should utilize summative evaluations, that include both quantitative and qualitative data, to build on the formative evaluation presented herein.

The identification of the core CFIR components of tele-GMH will help guide subsequent adaptations and expansion of the service to reach more older veterans who live in rural areas. In interviews, providers and leaders expressed the belief that referring providers, especially primary care providers, desire continuity care services for their patients with complex care needs. The core components of the models suggest that a combination of consultation care (to increase access to geriatric mental health expertise) and the availability of continuity care may form a base model.

Barriers emerged at multiple sites and included the challenge of fitting a new service into an existing infrastructure with ongoing telehealth operations. Site variability in these operations made implementation difficult at times, which is consistent with findings from other multisite geriatric telehealth programs (18). Leadership support was essential to support flexibility in workload expectations and productivity targets, given that some consultation-related work is not billable (e.g., conversations with referring providers, teams, and family members and extensive electronic health record reviews). For consultative and continuity services, the duration of and coordination needed for effective MH care delivery (e.g., technology support and speaking with caregivers) for older veterans with complex care needs can be daunting and reduce a provider's productivity. The challenges may be amplified for telehealth providers serving older patients, but they are consistently reflected as documented in a recent scoping review of telehealth implementation (19).

Key facilitators included planning implementation steps and being responsive by pivoting or changing an implementation strategy if needed (19). An example of this is designing a consultation template and revising it to improve the referring provider (user) experience. The leaders, implementation team members (tele-GMH providers), and local spoke site champions demonstrated that relationships are critical to a successful rollout process, mirroring findings from other VHA initiatives, including the integration of peer specialists into VHA primary care clinics (17) and the nationwide distribution of video telehealth tablets (20). Ongoing efforts by tele-GMH providers and CRH leaders to engage with referring providers and local stakeholders also fit with strong practices in telehealth implementation (19).

### Limitations

Our findings are limited by several factors. The interviews were conducted with a small number of CRH leaders and providers, which may not represent the perspectives of those at sites that have not developed tele-GMH care models to date. It is possible that other factors not specifically examined in the CFIR may be contributing to model adaptations and barriers to and facilitators to implementation. Despite the VA being an integrated, national healthcare system, there are many ways in which regional and local VA healthcare systems function independently. Given the differences in rurality and local infrastructure that guide model implementation, comparison between models to measure effectiveness is challenging. Future evaluations would benefit from more rigorous methods, including the collection of quantitative data (e.g., acceptance of referrals made to tele-GMH services and healthcare utilization metrics) alongside qualitative data. The four models described here are not expected to be the only models for tele-GMH care, as additional models may emerge. Further, these findings may not apply to non-VHA systems. Future research directions include defining the characteristics of tele-GMH care that may comprise a base model to facilitate future implementation and evaluation efforts. CFIR 2.0 (21) provides additional information that could inform a future implementation/evaluation study. For instance, the implementation process domain encompasses a number of steps that emerged from our interviews (i.e., assessing needs and context).

## Conclusion

The present evaluation described an initial tele-GMH service delivery model and its adaptations based on the characteristics of the locations where the model was implemented. Notably, all the models reflected the recently published Telehealth Principles and Guidelines for Older Adults (22) in their patient-centered approaches and emphasis on delivering services that fit with the larger system of care. Despite the variations among the models, the barriers and facilitators remained consistent, which may help standardize guidance for the future implementation of similar services.

## Data Availability

The datasets presented in this article are not readily available because due to privacy restrictions, the data, which are part of a quality improvement/program evaluation project, cannot be shared publicly. Requests to access the datasets should be directed to Christine Gould christine.gould@va.gov.
